# Adiponectin and leptin levels in HIV-infected patients with lipodystrophy in Southern India

**DOI:** 10.1186/1758-2652-13-S4-P75

**Published:** 2010-11-08

**Authors:** S Mini Jacob, K Annie Phoebe, R Hemalatha, MR Sivakumar, MP Ravindran, MV Natarajan

**Affiliations:** 1 The Tamilnadu Dr MGR Medical University, Department of Experimental Medicine, Chennai, India; 2 Ragas Dental College, Chennai, India; 3 Immunodiagnostics, Chennai, India; 4 The Tamilnadu Dr MGR Medical University, Chennai, India

## Purpose of the study

Evidence suggests that the level of adipokines (adiponectin and leptin) may be altered in lipodystrophy related to the long-term use of antiretroviral therapy (ART). The purpose of this study was to estimate the levels of adiponectin and leptin in HIV infected patients with lipodystrophy and to correlate them to metabolic parameters.

## Methods

In this cross sectional study, consenting 79 HIV+ve patients on antiretroviral therapy (for more than six months) visiting the Namakkal Government Hospital were recruited. Demography, anthropometry and ART regimens were collected. Patients' self-perception of lipodystrophy was determined using standardized questionnaires and clinically confirmed. An overnight fasting blood was drawn to determine serum adiponectin (Ray Biotech ELISA), serum leptin (DRG International ELISA) and insulin. Statistical analysis included analysis of variance and Pearson’s correlation.

## Summary of results

Men and women on ART with lipodystrophy (60.8%) when compared to those without lipodystrophy (30.2%) had similar mean adiponectin (p=0.842) moderately lower leptin (p=0.133), and higher insulin resistance (p= 0.031). Patients with lipodystrophy had lower BMI than those without lipodystrophy (p=0.02) and similar WHR (p=0.174).

Among the total study population stavudine usage was associated with lower adiponectin (p=0.018) but not leptin whereas insulin (p=0.007) and insulin resistance (p=0.00) positively correlated with leptin and not adiponectin. In lipodystrophic patients, adiponectin had positive correlation with BMI (p=0.014) and had no correlation with insulin (0.304), and insulin resistance (0.250) whereas leptin had positive correlations with insulin (p= 0.00) and insulin resistance (p=0.001). Among patients without lipodystrophy, adiponectin levels had negative correlation with stavudine usage (p=0.018) while leptin had no significant correlation.

## Conclusions

Patients with lipodystrophy had moderately lower leptin and higher insulin resistance compared to those without lipodystrophy. Leptin seemed to have influence on insulin and insulin resistance while adiponectin did not influence insulin levels in this study population. Stavudine usage influenced adiponectin but not leptin levels.

